# Application of multiple statistical tests to enhance mass spectrometry-based biomarker discovery

**DOI:** 10.1186/1471-2105-10-144

**Published:** 2009-05-14

**Authors:** Niclas C Tan, Wayne G Fisher, Kevin P Rosenblatt, Harold R Garner

**Affiliations:** 1 Department of Internal Medicine, Division of Translational Research, University of Texas Southwestern Medical Center, 5323 Harry Hines Blvd., Dallas, TX 75390, USA; 2 Departments of Biochemistry and Molecular Biology, Internal Medicine, University of Texas Medical Branch, 301 University Blvd., Galveston, TX 77555, USA

## Abstract

**Background:**

Mass spectrometry-based biomarker discovery has long been hampered by the difficulty in reconciling lists of discriminatory peaks identified by different laboratories for the same diseases studied. We describe a multi-statistical analysis procedure that combines several independent computational methods. This approach capitalizes on the strengths of each to analyze the same high-resolution mass spectral data set to discover consensus differential mass peaks that should be robust biomarkers for distinguishing between disease states.

**Results:**

The proposed methodology was applied to a pilot narcolepsy study using logistic regression, hierarchical clustering, t-test, and CART. Consensus, differential mass peaks with high predictive power were identified across three of the four statistical platforms. Based on the diagnostic accuracy measures investigated, the performance of the consensus-peak model was a compromise between logistic regression and CART, which produced better models than hierarchical clustering and t-test. However, consensus peaks confer a higher level of confidence in their ability to distinguish between disease states since they do not represent peaks that are a result of biases to a particular statistical algorithm. Instead, they were selected as differential across differing data distribution assumptions, demonstrating their true discriminatory potential.

**Conclusion:**

The methodology described here is applicable to any high-resolution MALDI mass spectrometry-derived data set with minimal mass drift which is essential for peak-to-peak comparison studies. Four statistical approaches with differing data distribution assumptions were applied to the same raw data set to obtain consensus peaks that were found to be statistically differential between the two groups compared. These consensus peaks demonstrated high diagnostic accuracy when used to form a predictive model as evaluated by receiver operating characteristics curve analysis. They should demonstrate a higher discriminatory ability as they are not biased to a particular algorithm. Thus, they are prime candidates for downstream identification and validation efforts.

## Background

The advent of mass spectrometry-based proteomic biomarker discovery augurs an increased output of diagnostic disease markers due to its ability to interrogate a complex constellation of proteins simultaneously. A typical proteomic biomarker discovery process comprises two major steps: data acquisition and data analysis. Data acquisition encompasses everything from sample collection, handling, and processing to the eventual production of mass spectra where proteins and peptides are represented as peaks with mass-to-charge (*m/z*) ratios and their corresponding signal intensities.

Technical issues pertaining to this step of the process are well-documented [1,2]. The ultimate goal is reproducibility of the mass spectra across replicates and the alignment of peaks across samples. To this end, next-generation mass spectrometers with high mass accuracy have been employed, along with efforts to standardize sample collection and processing protocols [3,4].

The data analysis phase of the process seeks to identify mass peaks that are differentially present between the groups of samples being compared. As with any expression data analysis, an array of pattern profiling systems exist that can reliably discover sets of classifying mass peaks. Unfortunately, a common and frustrating occurrence in proteomic biomarker discovery is the production of non-overlapping sets of biomarker peaks when different laboratories studying the same disease employ different statistical methods on the same data set. All data analysis methods have their strengths and weaknesses. The caveat lies in the realization of their statistical power only when applied to data sets where the underlying data distribution assumptions are met. As is often the case with mass spectrometry data, the data distribution is unknown.

Comparisons of different statistical methods on the same mass spectrometry data have been reported previously [5-7]. However, the ultimate goal of these reports was the selection of a method whose prediction model outperforms the rest of the methods under investigation when applied to a given set of experimental data and the subsequent recommendation of the method that prevailed for future analyses. This introduces bias in the selected marker peaks which are unique to a statistical method and are most often a result of overfitting. This is also true when peak reduction was performed using a predefined statistical method prior to submitting the remaining peaks for model building comparisons. In addition, a majority of these studies were performed using low-resolution mass spectrometer data with significant mass drifts across spectra within a single experimental run that further complicate analysis.

Therefore, in the proposed workflow, four unique statistical modeling approaches (parametric and non-parametric) are employed concurrently for the analysis of the raw peaks from the same high-resolution data set to obtain a set of consensus biomarkers. Consensus biomarkers are defined as mass peaks with discriminatory power between the groups being compared that end up on the list of differential peaks across at least two or more of the statistical strategies employed in the data mining analysis. The reasoning is that in lieu of the data distribution knowledge, mass peaks that survive stringent conditions across multiple statistical methods are more likely to be true "biomarkers" and not artefacts as a consequence of bias inherent to a particular algorithm. Convergence upon this distinct set of biomarkers using multiple analytical platforms will confer higher confidence in these markers as robust entities and will increase the chance that these markers may be adopted as diagnostic entities where subsequent identification and validation efforts should be directed.

The novelty of our approach lies in both the experimental design and the statistical evaluations. First, we used a mass spectrometer with high mass accuracy and low mass drift to generate high-resolution data, which is essential for accurate peak-to-peak comparison across spectra. Second, there was no biased peak selection prior to model building by the four statistical algorithms under investigation; all raw peaks within the 1,000 to 10,000 mass range were subject to each algorithm. Furthermore, to assure a fair comparison between the methods, the best discriminatory peaks from each method underwent the same diagnostic accuracy testing via receiver operating characteristic (ROC) curve analysis, as did the model consisting of only the consensus peaks. ROC confers a better sense of diagnostic performance of the biomarker peaks as it evaluates all possible cutoff values and produces the best trade-off between the rates of false-negative and false-positive results. In addition, the diagnostic accuracy measures will provide an indication of the clinical utility of the statistically significant differential peaks discovered.

This study has two objectives. The first is to perform biomarker data mining on the same data set using four different statistical platforms and compare the list of candidates from each platform to determine if the resulting peaks are specific to each method and whether it is at all possible to find peaks that are robust enough to exist across two or more platforms. The second objective is to group the consensus peaks and compare their diagnostic performance collectively against the best individual model from each statistical method. Our results reveal that even in the case of comparable performance between different statistical platforms, the model incorporating the consensus biomarkers across multiple platforms confers a more dependable set of peaks for further investigation.

## Results and Discussion

### Biomarker Selection

#### Logistic Regression

We used our modified, AIC-optimal logistic regression protocol to analyze the narcolepsy data set and compared the diagnostic power of the best model from this approach to the best model obtained using the default single-step calling of the PROC LOGISTIC in SAS. The diagnostic measures are shown in Table 1. The final model from the default stepwise procedure has a higher Akaike Information Criterion (AIC) statistic of 69.646 with two variables incorporated while the AIC-optimal model from the modified procedure has an AIC statistic of 57.798 with five variables incorporated. This means that the default stepwise model incorporated three predictors less than necessary to form a better predictive model. This is indicated by its lower Hosmer-Lemeshow goodness of fit statistic (0.669 for default versus 0.882 for AIC-optimal). The resulting default model also has a poorer discriminatory power than the AIC-optimal model as indicated by the lower area under the ROC curve. As for diagnostic accuracy, both models have comparable sensitivity, positive predictive value (PPV) and negative predictive value (NPV), but the default model lacks in specificity and the percentage of cases accurately predicted (Table 1). This demonstrates that the modified procedure performs better than the default in producing good predictive models, and was thus adopted in subsequent logistic regression analyses.

**Table 1:** Diagnostic accuracy measures from the default and AIC-optimal models in logistic regression

Logistic Regression Model	Default	AIC-Optimal
Number of variables in final model	2	5
Goodness of Fit	0.669	0.882
AIC Statistic	69.65	57.80
Area under ROC curve	0.793	0.910
Sensitivity (%)	63.16	57.89
Specificity (%)	82.22	95.56
PPV (%)	85.96	84.62
NPV (%)	84.09	84.31
Percent accuracy (%)	76.56	84.38

AIC = Akaike Information Criterion, ROC = Receiver Operating Characteristic, PPV = Positive Predictive Value, NPV = Negative Predictive Value

In the comparison between narcoleptic and non-narcoleptic samples, four optimal models were obtained, as listed in Table 2. The mass peaks from these four models were pooled as potential biomarkers selected from logistic regression.

**Table 2:** Discriminatory mass peaks from AIC-optimal models in logistic regression analysis on narcolepsy data set

Logistic Regression Model	1	2	3	4	Pooled
Number of variables	5	1	2	2	9

Mass peaks (*m/z*)	1431.80	1809.98	1809.98	1722.93	1431.80
	1839.98		3826.00	1740.94	1722.93
	2225.14				1740.94
	3986.99				1809.98
	5857.74				1839.98
					2225.14
					3826.00
					3986.99
					5857.74

#### Classification and Regression Tree (CART)

**Figure 1:**
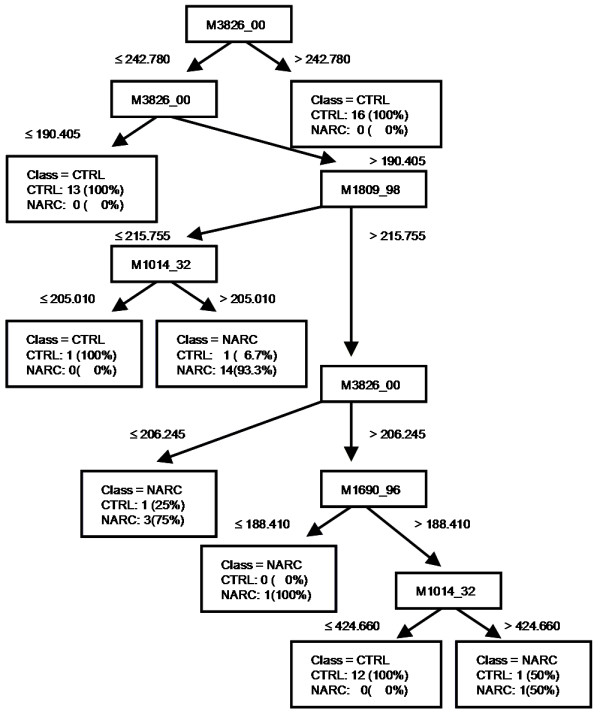
**Tree diagram of best model from CART analysis**.

The narcolepsy data set was analyzed using Ciphergen's Biomarker Patterns Software 5.0. Tree-building was first performed using the default Gini splitting criterion to obtain the best tree with the minimal cost. This was repeated with the remaining split criteria – Symgini, Twoing, Ordered Twoing, Class Probability, and Entropy. 10-fold cross-validation was used for testing. The best tree from CART is the tree with the lowest cost across all six splitting criteria. This optimal tree with a cost of 0.322 was obtained from the Twoing criterion and is shown in Figure 1. The diagnostic measures of this model are listed in Table 3.

**Table 3:** Diagnostic accuracy measures of optimal CART model

CART	Optimal Model
Number of variables in final model	6
Mass peaks (*m/z*)	1014.32, 1690.96, 1809.98, 3043.43, 3826.00, 3986.99
Area under ROC curve	0.984
Sensitivity (%)	78.95
Specificity (%)	88.89
PPV (%)	75.00
NPV (%)	90.91
Percent accuracy (%)	85.94

ROC = Receiver Operating Characteristic, PPV = Positive Predictive Value, NPV = Negative Predictive Value

#### T-test

In the t-test method, a peak is deemed differential if it is populated by statistically significant data points. The maximum p-value was set to 0.05 and the minimum signal intensity ratio was set to the default value of 1.5. Peaks that best distinguish the two groups under comparison were selected by increasing the stringency of the criteria by decreasing the minimal p-value or increasing the minimal fold-change. In this study, the default conditions were found to be optimal as decreasing the p-value below 0.05 and increasing the fold-change above 1.5 did not result in differential peaks. Only three possible candidate biomarkers were identified in the spectra – 1740.94 Da, 3598.07 Da, and 5078.90 Da. They were all higher in the narcolepsy samples. The diagnostic performance of this three-peak model is shown in Table 4.

**Table 4:** Diagnostic accuracy measures of optimal t-test model

T-test	Optimal Model
Number of variables in final model	3
Mass peaks (*m/z*)	1740.94, 3598.07, 5078.90
Sensitivity (%)	33.30
Specificity (%)	84.20
PPV (%)	50.00
NPV (%)	72.70
Percent accuracy (%)	67.90

All differential peaks have a p-value less than 0.05. PPV = Positive Predictive Value, NPV = Negative Predictive Value

#### Hierarchical clustering

Differential peaks from unweighted pair group method with arithmetic mean (UPGMA) clustering were first selected with a p-value < 0.05, followed by trimming of the remaining mass peaks based on fold-change between the two conditions under comparison. The three differential peaks obtained from this platform only had a fold-change of at least 10% (Table 5). Peaks that demonstrated a greater than 10% fold-change did not meet the p-value requirement. Their diagnostic accuracy measures were determined and are shown in Table 6.

**Table 5:** Statistically differential peaks from UPGMA model

Mass peak (*m/z*)	Fold change	p-value
1781.99	1.13	0.046
1809.98	1.15	0.007
3826.00	1.13	0.017

Peaks are presented with their respective fold change and p-value.

**Table 6:** Diagnostic accuracy measures of optimal UPGMA model

UPGMA	Optimal Model
Number of variables in final model	3
Mass peaks (*m/z*)	1781.99, 1809.98, 3826.00
Area under ROC curve	0.788
Sensitivity (%)	36.84
Specificity (%)	95.56
PPV (%)	77.78
NPV (%)	78.18
Percent accuracy (%)	78.13

ROC = Receiver Operating Characteristic, PPV = Positive Predictive Value, NPV = Negative Predictive Value

#### Consensus Peaks

Consensus peaks across at least two or more of the four platforms are defined as robust biomarkers. Even though the ideal scenario is to have consensus peaks across all four platforms, the two peaks that were considered truly robust in this study were mass peaks at *m/z *1809.98 and 3826.00 which were selected as statistically differential in three of the four approaches. No marker peaks appeared as differential across all four platforms. These two robust, consensus peaks were grouped collectively to form an independent diagnostic model. When used for diagnosis, these two peaks have a sensitivity of 63.16%, a specificity of 82.22%, a PPV of 85.96%, a NPV of 84.09%, and a percentage of cases correctly classified of 76.56%. The area under the ROC curve was 0.79 (Table 7).

**Table 7:** Diagnostic accuracy measures of consensus model

	Consensus Model
Number of variables in final model	2
Mass peaks (*m/z*)	1809.98, 3826.00
Area under ROC curve	0.793
Sensitivity (%)	63.16
Specificity (%)	82.22
PPV (%)	85.96
NPV (%)	84.09
Percent accuracy (%)	76.56

Consensus peaks included in this model are peaks selected as statistically differential across three of the four algorithms. ROC = Receiver Operating Characteristic, PPV = Positive Predictive Value, NPV = Negative Predictive Value

**Figure 2:**
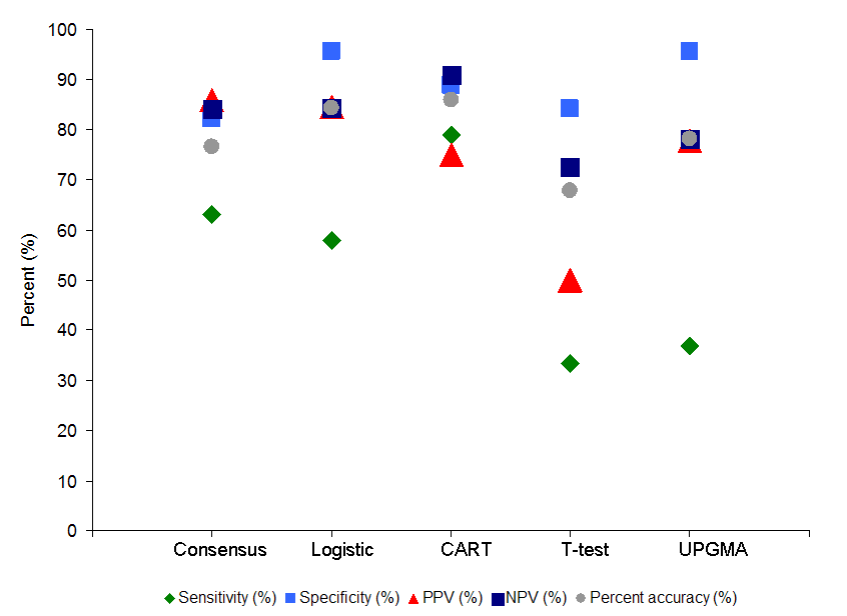
**Diagnostic measures comparison of consensus model to the best model from each of the four statistical approaches**. The sensitivity, specificity, positive predictive value (PPV), negative predictive value (NPV) and percent accuracy are plotted for the best model from each statistical approach. When the five parameters are evaluated collectively, the model with the best diagnostic performance is CART, followed by the Consensus model and Logistic Regression. The T-test and UPGMA models have a lower average diagnostic performance as evident in the greater spread of the values of the diagnostic accuracy measures.

In this study, UPGMA and the t-test produced predictive models that did not perform as well as those from logistic regression and CART, even though the model from UPGMA included the two consensus peaks. In contrast, the consensus model has the diagnostic potency in some diagnostic measures that is comparable to, if not better than, the individual models from each statistical platform (Figure 2).

Depending on the clinical applications (e.g. screening or confirmatory diagnostics), different diagnostic measures will take precedence. For example, high sensitivity is needed for screening but high specificity is needed for confirmation and subcategorization. Ideally, both need to be greater than 70% [8]. Sensitivity is a measure of how well the diagnostic test correctly identifies the disease cases whereas specificity is how well it correctly identifies the non-disease cases from the whole test population. Although admittedly limited in sample size, our comparison between narcolepsy samples versus all non-narcolepsy samples in this pilot study served to emulate general population screening, where these biomarkers are intended for. In this case, sensitivity is of more importance than specificity. Logistic regression suffers from low sensitivity (57.89%). CART prevailed in these measures with 78.95% sensitivity and 88.89% specificity, followed by the consensus model with a reasonable sensitivity of 63.16% and a specificity of 82.22%. This is very encouraging as the current genetic marker for narcolepsy in general is based on the presence of HLA DQB1*0602 which itself only has a specificity of 40% [9]. Genetic markers confer susceptibility but are not ideal disease biomarkers as most people who are positive for the HLA DQB1*0602 gene do not develop narcolepsy.

The more important diagnostic measures to consider are PPV and NPV, which evaluate the applicability of the diagnostic test on the target population. The precision of the test is measured by the PPV where a positive test reflects the probability that a subject will have the diagnosed condition (narcolepsy in this study). On the other hand, NPV reflects the probability that a negative test means the subject is disease free. The consensus model displays the highest PPV of 85.96% and an NPV of 84.09%, comparable to the best logistic model. CART has the highest NPV of 90.91% but lacks in PPV with only 75%.

To surmise, CART seems to produce the best model in this pilot study when all five diagnostic accuracy measures are considered collectively (Figure 2), followed by logistic regression. UPGMA and the t-test did not fair as well. Of interest is the performance of the consensus model which seems to be a good compromise between both CART and logistic regression. Albeit models from logistic regression and CART in this study performed better in a few of the diagnostic measures, not all peaks in those models warrant subsequent identification and validation efforts. This is because spurious peaks that are only specific to those models might be a reflection of overfitting and biases to the respective algorithm. These biases could be the reason why the logistic and CART models appear to perform better than the consensus model. Therefore, downstream validation efforts and resources will be better off directed at the consensus peaks.

An added advantage of the consensus model is the higher level of confidence in the true discriminatory traits of the peaks as they managed to survive various data distribution assumptions across statistical platforms to appear as statistically significant differential peaks. Another advantage of forming a consensus model is the trimming of the long list of potential biomarkers to be sequenced to the selected few with true discriminatory power. The ideal clinical assay will only need to focus on assaying the minimal number of biomarkers to accurately diagnose a disease state.

The methodology described here can be applied to any MALDI-TOF derived data set to reconcile the disparate potential biomarker mass peaks reported by different studies on the same disease, provided the same standard operating procedure is employed during data acquisition and the studies are significantly powered. This requires the incorporation of a sizeable sample set that is representative of the target population to impart confidence in the differential peaks discovered. A limitation of this approach is when there are no overlapping peaks across the statistical platforms used. However, when there are overlapping marker peaks, these consensus peaks will no doubt expedite efforts to identify robust biomarkers for clinical applications as their true discriminatory trait is reflected in their selection as differential biomarkers across several statistical platforms. Our laboratory is currently pursuing the identification of the consensus peaks via Fourier transform mass spectrometry.

## Conclusion

We have applied four distinct statistical approaches to the same high-resolution mass spectral data set from our narcolepsy study to discover mass peaks that are statistically differential. Even though each approach has its own assumptions on the data distribution of the data set, several of the mass peaks that have inherent discriminatory power appeared as potential biomarkers across platforms. In particular, peaks 1809.98 Da and 3826.00 Da were selected as discriminatory peaks across three of the four platforms and thus, deemed robust biomarkers.

When these two peaks were combined into a diagnostic model, they performed as well if not better than the individual models. They confer a specificity of 82% which is far greater than the current genetic marker HLA DQB1*0602 for narcolepsy in general. While we are aware we have analyzed only a limited number of patient samples thus far, our results are encouraging; this reiterates the promise that mass spectrometry-based biomarker discovery is capable of delivering potential biomarkers between disease states that can add on to the current battery of diagnostic tools, once validated in a larger sample size that is more representative of the screening population of course. Furthermore, with our methodology, consensus biomarkers with higher confidence in their ability to discriminate can be discovered and hence, should be the main candidates where downstream identification and validation efforts should be focused on to assess their suitability to be adopted in a diagnostic assay or as therapeutic targets.

## Methods

**Figure 3:**
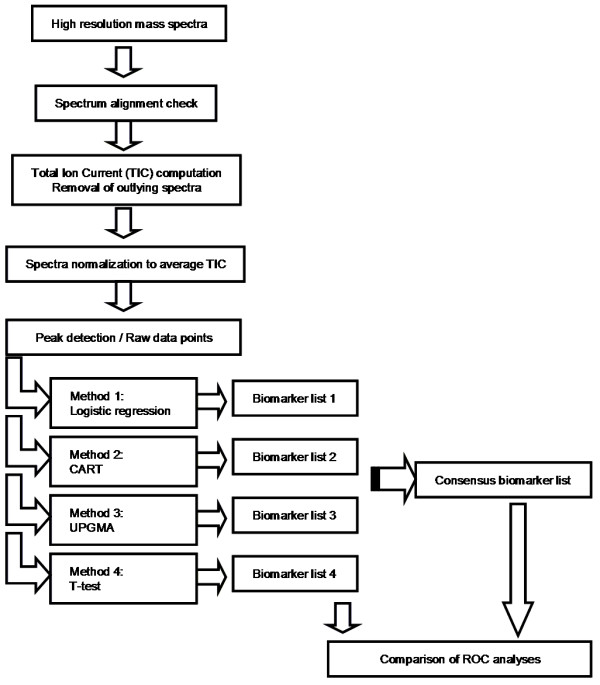
**Schematic diagram of the multi-statistical workflow to discover consensus biomarker peaks**.

The method described here is intended for biomarker discovery based on high-resolution matrix-assisted laser desorption/ionization time-of-flight (MALDI-TOF) mass spectrometry proteomic data. A flowchart depicting the methodology is shown in Figure 3.

### Mass spectral data

The methodology described here can be applied to any MALDI-TOF derived data set for any disease, provided the same standard operating procedure (from biological sample procurement, processing, and complexity reduction to actual mass spectrometry data acquisition) is employed. We chose to demonstrate this analysis process on narcolepsy, a disease of interest to some of the authors, and for which every step from sample processing to data acquisition has been conducted by the group.

Narcolepsy is a neurological disorder known to affect sleep states. It is estimated to affect 3 million people worldwide and 200,000 people in the United States (prevalence of 1:2000). Studies have shown that narcolepsy is a result of defective hypocretin transmission due to selective damage to hypocretin neurons [10,11]. The current diagnosis is symptom-based which can result in false positives [12]. Discovery of novel biomarkers will enable early detection of narcolepsy either before or at the beginning of irreversible neuronal loss.

The mass spectral data set used in this pilot study was generated from 30 serum samples from narcoleptic and non-narcoleptic patients, courtesy of Dr. Emmanuel Mignot from the Stanford Center for Narcolepsy. The narcolepsy samples were obtained from patients diagnosed with narcolepsy and are positive for the HLA DQB1*0602 susceptibility gene. The control samples were obtained from patients not diagnosed with narcolepsy and are either positive or negative for the HLA DQB1*0602 susceptibility gene. Narcolepsy diagnosis was confirmed by measuring the level of hypocretin in the patient's cerebrospinal fluid.

All samples were stored at -80°C before analysis. In an effort to simplify the complexity of the serum proteome, only the cargo bound to the carrier protein albumin was analyzed. Briefly, serum albumin was selectively captured, followed by the elution of the bound protein and peptide fragments using ProXPRESSION Biomarker HT Enrichment Kit (PerkinElmer). These fragments were then applied onto a Bio-Rad ProteinChip with IMAC capture surface charged with nickel. Unbound species were washed away before the bound species were introduced to the mass spectrometer. Mass spectra were acquired on a high-resolution prOTOF 2000 MALDI-OTOF Mass Spectrometer (PerkinElmer/SCIEX). The orthogonal design allows the use of a single external mass calibrant to achieve better than 10 ppm mass accuracy from 1–10 kDa. In this study, a 2-point external calibration of the prOTOF instrument was performed before acquiring the spectra in a batch mode from 96 sample wells. A total of 30 serum samples were run in triplicate to measure and enhance the reliability of MALDI protein profiling [13]. Each spectrum contains on the average 1 million data points. The narcolepsy mass spectral data set is available upon request.

### Data preprocessing

**Figure 4:**
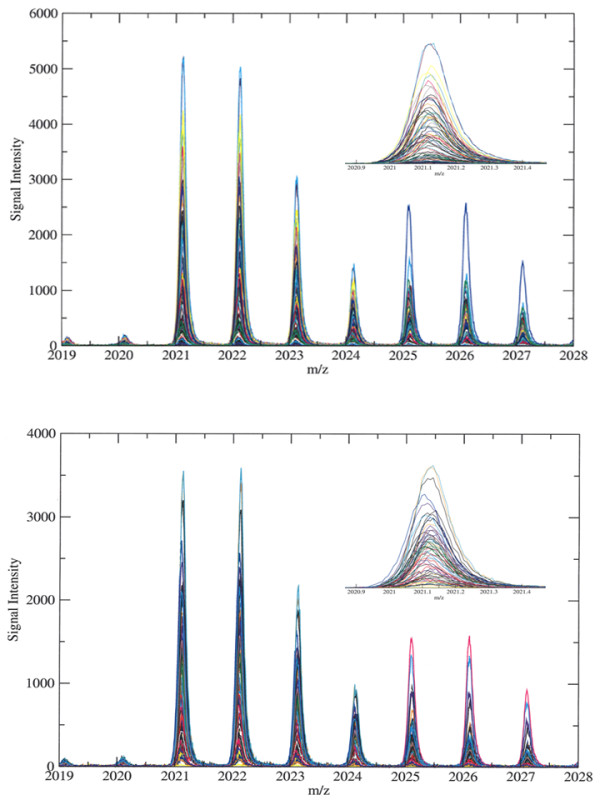
**Peak alignment across spectra**. Representative spectra from the high performance prOTOF mass spectrometer are shown before (top panel, 84 spectra) and after (bottom panel, 68 spectra) data preprocessing. Data preprocessing entailed removal of outlying spectra and normalization of signal intensity to the average TIC. Shown in the insets are the enlarged view of peak at *m/z *= 2021 which has a mass accuracy of < 10 ppm, rendering spectrum-to-spectrum alignment unnecessary. Each spectrum is plotted in a different color through arbitrary assignment.

Two serum samples that appeared reddish were noted and may have been contaminated with cellular components (e.g. hemoglobin from hemolysis), so their spectra were omitted from the study. The spectrum-to-spectrum alignment was checked for 6 different peaks across the *m/z *range of 1–10 kDa and found to be acceptable (< 10 ppm). Figure 4 shows peak alignment across representative spectra before and after preprocessing. In order to enhance the reproducibility of the peaks in the profiles generated, all of our samples were processed and analyzed on the same day, in one sitting to minimize inter-day variation of the peak intensities. In our study, the coefficient of variation of peak intensities was 5–10%, similar to those reported by Albrethsen [2]. Due to the high mass accuracy and minimal mass drift of the prOTOF [2,4,14], no further spectral alignment was necessary.

The total ion current (TIC) of each spectrum was calculated and the average TIC was computed across all 84 spectra. 16 spectra with a TIC value that is either twice or half of the average TIC were deemed outliers and were omitted from the study. Global normalization of the signal intensity of the mass peaks was performed by normalizing to the average TIC of the remaining 68 spectra. This confers a sense of commonality across spectra for statistical comparisons. All spectra were run through the Progenesis PG600 software (Nonlinear Dynamics, UK) for peak detection using the following parameters to remove background noise: noise filter size 4, background filter size 70, and isotope detection in MALDI mode with peak threshold 25 and window 0.1 Da. Noise filtering was performed using the Savitsky-Golay smoothing algorithm. The background correction was performed using a baseline adjustment method which relies upon a 'top-hat' morphology operator as a filter for contrast enhancement. The peak threshold discards peaks under the preset size. Both the peak threshold and window size allow for the detection of monoisotopic peaks within 0.1 Da.

An inherent challenge in analyzing mass spectral data is that they suffer from high dimensionality, and chemical and biological noise [15]. Therefore, in an attempt to reduce dimensionality, the *m/z *peak list was trimmed and only peaks that fall within the *m/z *range 1–10 kDa were subject to statistical analysis. We chose the lower cutoff value of 1 kDa to exclude any potential chemical noise contributed by the ionizing matrix. The upper cutoff value of 10 kDa was selected because the ionization efficiency of molecules decreases with increasing mass and few peaks above the noise level are detected beyond this value. This resulted in 247 peaks that were subject to each classification strategy, except for the t-test method which relies on data points and not individual peaks.

### Biomarker selection

To date, no guidelines exist that facilitate the selection of appropriate statistical methods to employ for data analysis in mass spectra. The choice of statistical platform for each study remains vastly heuristic and subjective. This is because the hunt for differential biomarkers in mass spectra is a data-driven process and no *a priori *knowledge of data distribution is available. The situation is made worse by the absence of a particular statistical method that has proven superior over others in its ability to handle the problem of high dimensional data. Consequently, various learning algorithms have been engaged in the field for classification purposes, each with its underlying biases and assumptions of distribution [16-18].

In our methodology, we have adopted both parametric (logistic regression, hierarchical clustering, t-test) and non-parametric (CART) approaches to analyze our MALDI-TOF-derived data set. These four statistical platforms were selected because they were available to us either as licensed softwares that accompanied our mass spectrometers or were developed in-house. The maximum p-value was set to 0.05 across all platforms to obtain statistically significant marker peaks. Each statistical approach was applied to the same mass spectral data set using its default parameters to capitalize on the strengths of each approach in discovering the best differential peaks based on the data distribution assumptions of that particular algorithm. This is essential as our reasoning is to impart confidence on the consensus peaks that are able to survive differing data distribution assumptions as reflected in the individual classification method.

In parametric approaches, the data are assumed to originate from variables with a certain probability distribution (such as normality and homoscedasticity). Non-parametric approaches are more robust and yield greater power with less well-behaved data since no prior assumptions are made. Therefore, mass peaks that are robust enough to survive both approaches and appear as potential biomarkers are then deemed to possess true discriminatory power, regardless of the distribution assumptions.

### Logistic regression

Logistic regression is an additive, parametric modeling technique that can be used to estimate the probability of an individual acquiring a disease [19]. It produces the most parsimonious model (incorporating the minimum number of variables necessary) to explain the observations or to categorize the disease and control groups. Logistic regression does not require a normal distribution and homoscedasticity for the outcome variable. Instead, it assumes the outcome has a binomial distribution and is governed by the logistic function. An advantage of modeling using logistic regression is its ability to estimate the associated risk with each variable. A drawback is that logistic regression suffers from the inability to accurately estimate the needed parameters when the two groups are perfectly separated based on the variables included in the model. This situation, however, will not be encountered in CART as discussed below. Furthermore, proteomic data provide a large number of variables (peaks) relative to observations from the few, clinical samples available in most studies. This results in the problem of sparse data [15] which could lead to model instability from overfitting and inaccurate estimates of the classification parameters.

To address this issue, a more detailed nine-step protocol was implemented based on recommendations by SAS User Group International (SUGI) [20,21] and the Environmental Protection Agency [22] in lieu of the default one-step calling of the PROC LOGISTIC procedure in SAS.

All of the variables (*m/z *values representative of peaks) from the preprocessed data set are first run through a univariate analysis to test for significance in predicting the outcome of the samples. These variables are then checked for correlation. Since logistic regression assumes no collinearity among the variables, each pair of correlated variables will have the less significant one removed based on the univariate analysis. This is necessary to reduce the number of variables per observation. In addition, variables with a Variation Inflation Factor (VIF) exceeding 10, indicating multicollinearity were also removed [23].

Modeling is performed using a stepwise procedure, where variables are added and/or removed at each step depending on the significance level for entering (SLENTRY = 0.990) and staying (SLSTAY = 0.995) in the model. This procedure continues until no variables can be added or removed. The stepwise technique effectively reduces the number of models under consideration while the less stringent entry and stay criteria allow more variables to be considered concurrently. The model with the lowest Akaike Information Criterion (AIC) score will indicate the optimal number of variables (n) to be incorporated into the model. AIC was preferred over the Schwarz Information Criterion (SC) that also accompanies logistic regression in SAS because it is better suited for the current goal of prediction [20]. Subsequent modeling with the best subsets selection method will then produce a list of potential models incorporating n-2, n-1, n, n+1, and n+2 variables. The best subset selection method was coupled to the AIC analysis to incorporate suboptimal models that flank the optimal model with the lowest AIC. Only models with a high Hosmer-Lemeshow (Goodness of Fit) score and a low AIC score will be retained. They then undergo diagnostic checking to identify outlier observations and interactions between variables. Finally, ROC curve analysis is performed on the few surviving models. Peaks from these models collectively create a pool of potential biomarker candidates. Logistic regression was performed using the Statistical Analysis Software (SAS) (SAS Institute Inc., Cary, NC).

### Classification and Regression Tree (CART)

CART is non-parametric and non-algebraic, and is a form of binary recursive partitioning where each group of patients as described by their spectra at each "node" in a decision tree can only be split into two groups [24]. The construction of the classification tree begins with the variable that maximizes the group homogeneity of the daughter nodes. This process is then repeated where every daughter node is split into two subgroups until all variables have been exhausted or the end nodes are homogeneous. Variable selection at each node is performed with one of six criteria – Gini, Symgini, Twoing, Ordered Twoing, Class Probability, or Entropy using Ciphergen's Biomarker Patterns Software (BPS).

Since it is non-parametric, no assumptions are made about the underlying distribution of the variables and being less sensitive to outliers, highly-skewed, non-normal data sets can be handled. A drawback is that some models can be unstable. Since all possibilities are evaluated at each splitting node, there is the potential of overfitting the model. To account for this, the tree is then pruned back using 10-fold cross-validation to obtain the optimal tree with the lowest average decision cost or error rate.

### T-test

A high-throughput software pipeline that uses a t-test to analyze mass spectrometry data was also applied to the data set. This analysis is similar to a previously published method [4] but uses a t-test instead of the Cohen's d statistic used in the published method. The method using either the t-test or d statistic yield very similar results. This software is a parametric, non-algebraic method for finding differentially expressed peaks by applying three filters to the average intensity value of each raw *m/z *data point between the two groups being compared. The first criterion uses a t-test for measuring the difference between the means. Typically, two signals whose means differ with a p-value of 0.05 or less are deemed significantly different. The second criterion requires the signals to be above the noise level. The third criterion requires the ratio between the two signals to be above a preset threshold (a so-called fold change determinant). Signals that pass all three filters suggest that a difference exists between the two groups being compared.

An advantage of this approach is that it does not require peak finding and thus is applicable to spectra with overlapping or non-Gaussian peaks, conditions that would confound most peak finding algorithms. Further, the method automatically provides a weighting factor for each peak, as peaks that differentiate the most have more discriminating data points on them [4]. The differential peak used in model building corresponds to the monoisotopic peak. This software is described in detail in [4] and is available upon request.

### Hierarchical clustering

Hierarchical clustering analysis calculates the dissimilarity, called the distance, between the individuals. The hierarchical clustering method used was the unweighted pair group method with arithmetic mean (UPGMA) algorithm that is based on the average distance between the clusters and their correlation. UPGMA is a parametric technique that is most commonly used in microarray [25,26] and mass spectrometry data analysis [16] because no prespecification of number of clusters is required. The resulting discriminant markers between the two groups are dictated by the parameters for biomarker selection, such as minimum peak intensity and p-value. This ensures we report only the peaks that are above the noise level and are statistically significant. Each group is compared and a p-value is automatically calculated for each peak using ANOVA based on the spectra groups. The tabular data is filtered by p-value and minimal fold-change to obtain the most significant peaks. UPGMA clustering was performed on the Progenesis PG600 software (NonLinear Dynamics, UK).

### Receiver operating characteristic (ROC) curve analysis

The performance of the discriminatory peaks in the resultant models from logistic regression and UPGMA hierarchical clustering was evaluated via ROC analyses using SAS. The diagnostic accuracy measures of interest are the sensitivity, specificity, positive predictive value (PPV), negative predictive value (NPV), prediction accuracy, and the area under the ROC curve for narcolepsy classification. These parameters were obtained from the CART models via 10-fold cross-validation using the BPS software, and from the t-test models based on the distance proximity of the differential data points from the spectrum to be classified to those from spectra with known classification [4].

## Abbreviations

AIC: Akaike Information Criterion; CART: classification and regression tree; HLA: human leukocyte antigen; IMAC: immobilized metal affinity chromatography; MALDI-TOF: matrix-assisted laser desorption/ionization time-of-flight; NPV: negative predictive value; PPV: positive predictive value; ROC: receiver operating characteristic; SC: Schwarz Information Criterion; TIC: total ion current; UPGMA: unweighted pair group method with arithmetic mean; VIF: variation inflation factor.

## Authors' contributions

NCT and KPR conceived the laboratory experiment. NCT, KPR and HRG conceived the basic analytical approach. NCT performed the experiments, performed all analyses except for the t-test analysis and wrote the manuscript. WGF performed the t-test analysis. All authors read and approved the final manuscript.
